# PGK1: A Common Biomarker and Therapeutic Target Linking Sarcopenia and Osteoporosis Through Fibroblast‐Mediated Pathways

**DOI:** 10.1049/syb2.70037

**Published:** 2025-10-13

**Authors:** Kun Zhang, Hailong Li, Xinhong Chen, Ping Tang, Meng Wang, Chunting Yang, Rong Su, Xiaqin Gao, Fan Zhang, Juan Han

**Affiliations:** ^1^ Department of Trauma Orthopedics Gansu Provincial Traditional Chinese Medicine Hospital Lanzhou China; ^2^ Department of General Practice Luohu Clinical College School of Medicine Shantou University Shenzhen China; ^3^ Department of Geriatrics Affiliated Hospital of Gansu University of Traditional Chinese Medicine Lanzhou China; ^4^ Department of Internal Medicine First School of Clinical Medicine Gansu University of Chinese Medicine Lanzhou China; ^5^ Department of Geriatrics Shenzhen Integrated Traditional Chinese and Western Medicine Hospital Shenzhen China; ^6^ Department of Pediatrics Gansu Provincial Traditional Chinese Medicine Hospital Lanzhou China

**Keywords:** big data, bioinformatics, data analysis, molecular dynamics method, network analysis, signal transduction

## Abstract

Sarcopenia and osteoporosis share pathophysiological links, but their co‐occurrence mechanisms remain unclear. This study aimed to identify molecular mediators of their co‐development using bioinformatics. Datasets for sarcopenia (GSE56815) and osteoporosis (GSE9103) were retrieved from GEO. Differentially expressed genes (DEGs) were analysed via edgeR and limma. Gene ontology (GO), Kyoto encyclopaedia of genes and genomes (KEGG) and weighted gene co‐expression network analysis (WGCNA) identified shared pathways and hub genes. Protein–protein interaction (PPI) networks were constructed using STRING and Cytoscape. We validated hub genes in independent datasets (GSE13850, GSE8479) and assessed via ROC curves. Immune infiltration, single‐cell analysis and drug prediction were performed. We identified 134 common DEGs (30 upregulated, 104 downregulated). WGCNA and PPI analysis revealed 14 hub genes (APOE, CDK2, PGK1, HRAS, RUNX2 etc.), all with ROC‐AUC > 0.6. PGK1 was consistently downregulated in both diseases and linked to 21 miRNAs and six transcription factors (HSF1, TP53, JUN etc.). Single‐cell analysis localised PGK1 predominantly in skeletal muscle fibroblasts. DrugBank identified lamivudine as a potential PGK1‐targeting therapeutic. PGK1 emerged as a central downregulated gene in sarcopenia and osteoporosis, enriched in fibroblasts and modulated by lamivudine. These findings highlight PGK1 as a shared diagnostic and therapeutic target, offering insights into musculoskeletal crosstalk.

## Graphical abstract

A bioinformatic analysis identified 14 key genes, including APOE, CDK2, PGK1 and others, that are significantly associated with both sarcopenia and osteoporosis, with all showing an area under the curve > 0.6 for both conditions. PGK1 was confirmed as a central hub gene that is strongly down‐regulated in both sarcopenia and osteoporosis. Additionally, LAMIVUDINE was identified as a small molecule targeting PGK1, and single cell analysis revealed that PGK1 is primarily enriched in fibroblasts within skeletal muscle.

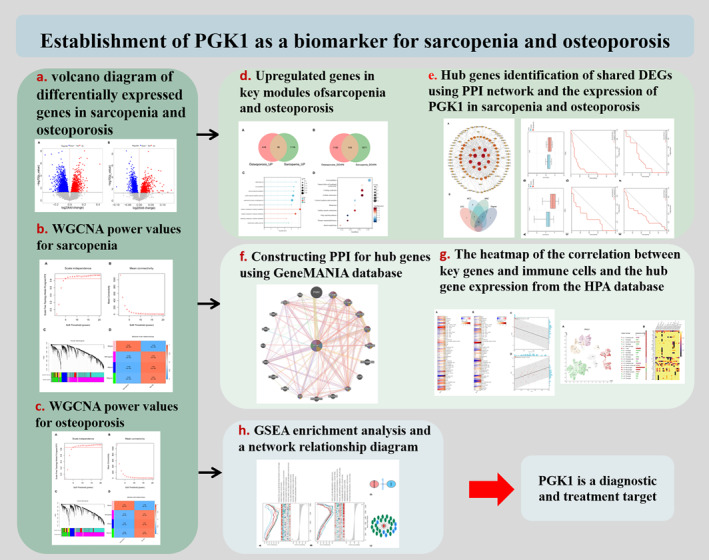

## 1 Introduction

Primary osteoporosis is a bone metabolic disease related to age and hormone levels, and increasingly, its harmful effects have been gradually realised and evaluated. However, early diagnosis and prevention of osteoporosis are often delayed due to its hidden onset, lack of unique symptoms and patients’ inability to seek urgent medical treatment. Notably, with the advent of ageing globally, the number of people suffering from osteoporosis has grown huge, with a concerning tendency towards younger people. Similarly, Sarcopenia is defined as an age‐related decline in skeletal muscle mass and function, specifically characterised by a decrease in the number and function of muscle tissue. Importantly, these two conditions share a common pathogenesis and interact with each other, a phenomenon defined as osteomyopenia [1] and dyskinesia syndrome [2]. Collectively, many chronic musculoskeletal problems can be caused by osteoporosis and sarcopenia, which are now recognised as key risk factors for adverse events such as falls, fractures and mobility difficulties in elderly people. These complications increase the risk of disability and mortality. Thereby [3], seriously affecting patients’ physical health and quality of life while increasing the economic burden and massively consuming medical resources. Given these impacts, research on the correlation between the occurrence and development of sarcopenia and osteoporosis is currently a hot research topic.

To clarify, osteoporosis is a systemic bone disease characterised by low bone mass, destruction of the microstructural deterioration, whereas sarcopenia is a systemic progressive loss of skeletal muscle mass and function that typically occurs with age, resulting in increased bone fragility and susceptibility to fractures [1]. Structurally, there are three types of osteoporosis: primary, secondary and idiopathic. Specifically, primary osteoporosis includes postmenopausal osteoporosis (type I) and elderly osteoporosis (type II), whereas secondary osteoporosis is caused by a range of factors such as endocrine and metabolic system diseases, connective tissue diseases, disorders of the kidney and digestive system as well as physical and chemical factors such as drugs and alcohol [4]. Functionally, the musculoskeletal system maintains the structure and framework of the body, with tissues such as bones, muscles, tendons and ligaments interconnected to form joints, thereby supporting the body for movement under the control of nerves. Moreover, the skeletal and muscular systems not only have endocrine functions but also regulate other tissue functions, coordinate and interact with other tissues and maintain homoeostasis and physiological functions.

Sarcopenia is a systemic and progressive disorder characterised by age‐related loss of skeletal muscle mass and function, and is associated with decreased mobility, falls, fractures and death [2]. Importantly, in recent years, there has been further research on sarcopenia, which has led to forming a new consensus, revising diagnostic criteria, thereby improving the diagnosis and risk awareness of sarcopenia, and delaying disease development through early detection, prevention and treatment. Mechanistically, changes in the muscle tissue structure and hormone imbalance, combined with external factors such as insufficient energy intake, were shown to contribute to the occurrence and development of sarcopenia [5]. Moreover, ageing, lack of exercise, malnutrition, smoking, excessive sleep and diabetes are all associated with the onset of oligomyositis [6]. Notably, genetic factors, nutritional imbalances, mechanical loads, cytokines and biological mechanisms that regulate hormone levels are associated with the occurrence and development of sarcopenia and osteoporosis. Recent breakthroughs in studies have shown that peroxisome proliferators activate receptor‐γ‐encoding genes, particularly coactivating factor‐1a, myocardial cell enhancing factor 2C, methyltransferase‐like protein 21C, co actin‐3, muscle growth inhibitor and glycine‐N‐acyltransferase, which are specifically associated with the pathogenesis of sarcopenia and osteoporosis [7]. Clinically, as the metabolic function of elderly people decreases, their ability to absorb various nutrients correspondingly weakens, rendering them prone to insufficient intake and absorption of nutrients, such as proteins, vitamins and calcium. Specifically, long‐term lack of raw protein for synthesising bone matrix can lead to delayed bone formation, accompanied by gradual atrophy of muscle cells, and a decrease in the quantity and quality of skeletal muscle. Furthermore, low blood calcium levels can subsequently lead to secondary hyperparathyroidism, triggering increased secretion of parathyroid hormones, enhanced bone metabolism and absorption and destruction of the bone. Additionally, vitamin C deficiency significantly reduces the synthesis of hydroxyproline in the bone matrix, thereby affecting the normal growth of the bone matrix and the ability of bone cells to synthesise and secrete alkaline phosphatase. Intriguingly, normal gut microbiota have been found to play an important role in the absorption of intestinal nutrients, ultimately affecting skeletal muscle metabolism through various pathways and affecting skeletal and muscle function [8]. Building upon these findings, Based on the GEO database, building upon these findings, this study aims to uncover commonly expressed genes in patients with osteoporosis and sarcopenia by analysing the GEO database, with the ultimate goal of constructing a risk score model to mine the prognostic characteristics of new common genes for these co‐occurring conditions. Ultimately, the purpose of this investigation is to develop new prognostic indicators for diagnosis and treatment while revealing prospective therapeutic targets and providing novel insights into the mechanisms and functions of commonly expressed genes in the development of osteoporosis and sarcopenia.

## 2 Materials and Methods

### 2.1 Data Acquisition

The datasets used in this analysis were downloaded from the NCBI GEO database (https://www.ncbi.nlm.nih.gov/).

### 2.2 Data Preprocessing and Gene Annotation

First, the expression data were downloaded from the GEO database, preprocessed, standardised and transformed into a probe expression matrix in log2 form. Second, the platform annotation file was downloaded, and probes that did not match gene symbols were removed through one‐to‐one matching of probe numbers and gene symbols. For different probes mapped to the same gene, the mean value of the different probes was taken as the final expression value of this gene.

### 2.3 Differential Gene Identification

The Limma package (version 3.10.3, http://www.bioconductor.org/packages/2.9/bioc/html/limma.html) was used to generate differential gene expression. The classic Bayesian method is based on the training set for osteoporosis and sarcopenia, and differential gene expression analysis was performed for the two diseases compared to normal. After analysing all genes, the corresponding *p* values were obtained. Genes with a differential expression threshold (*p* < 0.05) were selected as differentially expressed genes.

### 2.4 WGCNA Screening Disease Related Module Genes and Identification of Co Susceptible Genes

Based on the differential gene expression levels in the training set for osteoporosis and sarcopenia, the R package WGCNA (version 1.61, https://cran.r‐project.org/web/packages/WGCNA/) was used to analyse the input genes to identify highly synergistic gene set modules. In the WGCNA algorithm, the elements in the defined gene co‐expression matrix are the weighted values of the correlation coefficients of genes, and the weights are chosen such that the connections between genes in each gene network follow a scale‐free network distribution. Here, the weighted value represents the soft power threshold. First, by setting a series of power values, the square value of the correlation coefficient between the connectivity *k* and *p* (*k*) under each power value and the average connectivity was calculated. Appropriate power values are then selected to ensure that the connectivity between the genes in the network follows a scale‐free network distribution. Second, based on clustering and dynamic pruning methods, parameters (min Module Size = 30: each module contains at least 30 genes; merge CutHeight = 0.25: the similarity coefficient when merging modules is 0.25) were set to aggregate highly correlated genes into modules. Finally, the correlation between the module and phenotype was calculated; the phenotype refers to sample group classification and the modules that are positively correlated with enrichment scores and with a *p* value less than 0.05. Based on the results of WGCNA, the module genes related to the two diseases were intersected to obtain common pathogenic genes.

### 2.5 GO and KEGG Pathway Analysis

Based on the common disease‐related genes, gene ontology and KEGG pathway enrichment analyses were performed on the differentially identified disease‐related genes using the R package cluster Profiler, and *p* < 0.05 was considered a significant enrichment result.

### 2.6 PPI Network Construction and Hub Gene Identification of Shared DEGs

Database prediction analysis was performed using STRING (Version 10.0, http://string‐db.org/) to determine whether there was an interaction relationship between proteins encoded by common differentially expressed genes. The input gene set was the common differential gene obtained above, and the species was *Homo sapiens*. The PPI score cutoff was 0.9, and all protein nodes involved in interactions were included in the genes mentioned above. After obtaining the PPI relationship pair, the CytoHubba plugin (Version 0.1, http://apps.cytoscape.org/apps/CytoHubba) was used to perform the following topological property analysis on the PPI network nodes constructed above, which includes MCC, MNC, degree and EPC. The top 20 genes for each topological property ranking were then selected, and the intersecting genes were hub genes.

### 2.7 Efficacy Evaluation of Hub Gene

For the key genes obtained in the early stage, the expression distribution in the validation sets of the two diseases was displayed using box plots, and the differences were tested using limma packages. Genes with significant test results and consistent up‐ and downregulation were selected as the final hub genes. The pROC package, Version 1.12.1, in the R language (https://cran.r‐project.org/web/packages/pROC/index.html) was used to draw ROC curves for all datasets (training and validation sets) to evaluate the accuracy of the diagnostic features.

### 2.8 PPI Construction of Hub Gene

GeneMANIA database (https://genemania.org/) PPI analysis was conducted on the final hub gene and its interacting genes to predict colocalisation, shared protein domains, co‐expression, prediction and correlation between pathways.

### 2.9 Evaluation of Immune Cell and Stromal Cell Infiltration

The R package xCell (version 3.8, https://github.com/dviraran/xCell) was used to conduct cell type enrichment analysis on stromal cell types and 64 immune cell types, and Spearman correlation analysis was performed to investigate the correlation between hub genes and different cells, and the results were displayed in the heat map.

### 2.10 Validation of Hub Gene Expression in HPA Database

The expression profiles of hub genes and recognisable cell‐type markers in different single‐cell cluster types were obtained from the HPA database (https://www.proteinatlas.org/). Pearson’s method was used for correlation analysis, and a *p* value < 0.05 was statistically significant.

### 2.11 Single Gene GSEA Analysis

The gene set enrichment analysis (GSEA) algorithm was used to identify differential regulatory pathways between high‐ and low‐expression groups targeting hub genes to determine the activated or suppressed signalling pathways in the disease, with a significant enrichment threshold set at *p* < 0.05.

### 2.12 Regulatory Relationship Between TF Gene and miRNA Gene Co Expression Network

The hub genes in the gene module were run to obtain a list of predicted miRNAs target regulatory relationship pairs in miRWalk 3.0 (http://mirwalk.umm.uni‐heidelberg.de/) and then run to obtain a list of predicted miRNAs target regulatory relationship pairs to construct a miRNA regulatory network. Subsequently, the Jaspar database (https://jaspar.genereg.net/) was used to accumulate hub genes to predict transcription factors and construct a TF target miRNA network using Cytoscape software.

### 2.13 Prediction of Small Molecule Drugs and Molecular Docking Analysis

The DrugBank database (https://go.drugbank.com/) was used to predict drugs that have an interaction relationship with the hub gene and to observe the interaction relationship between drugs and genes. The last step was to employ molecular docking to determine the binding capability of potential small molecule drugs with PGK1. The 3D structures of the active components (ligands) were extracted from the PubChem database in SDF format. The PGK1 receptors were selected using PDB files from the Protein Data Bank (https://www.rcsb.org/). The ligand and protein files were then sent to the CB‐Dock2 platform for preliminary molecular docking (https://cadd.labshare.cn/cb‐dock2).

## 3 Results

### 3.1 Identification of Differential Genes

**FIGURE 1:**
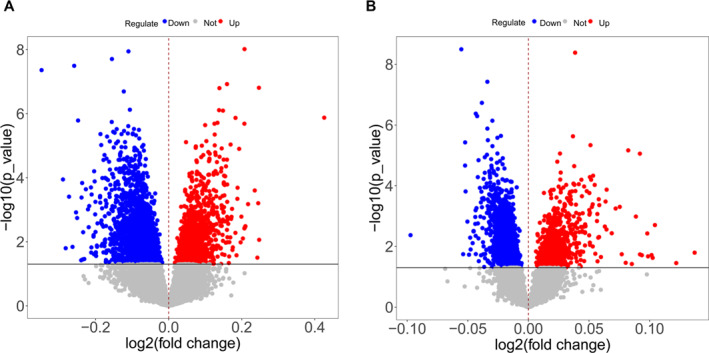
The volcano diagram of differentially expressed genes in sarcopenia and osteoporosis. (A) The volcano diagram of differentially expressed genes in sarcopenia (red represents upregulated genes, blue represents downregulated genes and grey represents insignificant gene differences); a total of 1289 upregulated and 2393 downregulated genes were identified in sarcopenia (log2FC > 1, *p* < 0.05). (B) The volcano map of differentially expressed genes in osteoporosis (red represents upregulated genes, blue represents downregulated genes and grey represents insignificant gene differences) shows a total of 782 upregulated and 1691 downregulated genes were identified in osteoporosis.

According to the threshold set by the method, 1289 upregulated and 2393 downregulated genes were identified in sarcopenia. Osteoporosis resulted in 782 upregulated genes and 1691 downregulated genes (log2FC > 1, *p* < 0.05). Based on the screened differential genes, a volcanic map was drawn, as shown in Figure 1A,B.

### 3.2 WGCNA Screening Disease Related Module Genes

**FIGURE 2:**
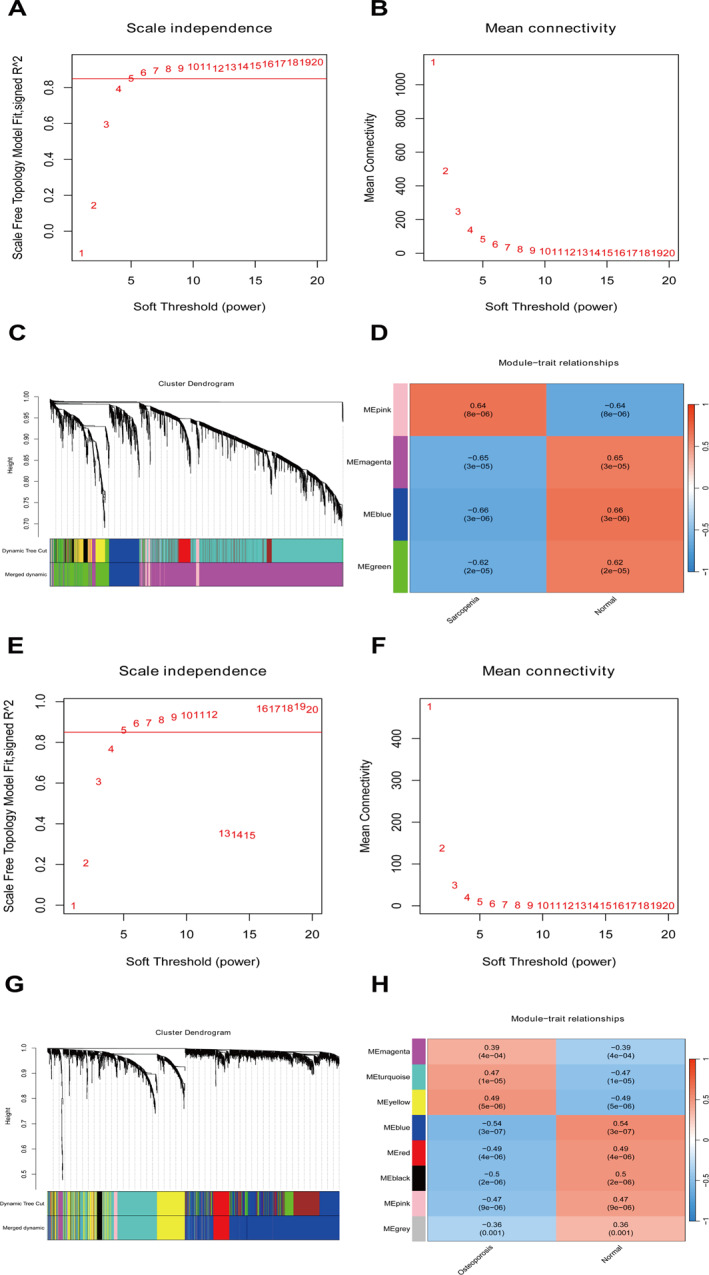
The results of WGCNA power values for sarcopenia. (A) Soft threshold (power) represents weight, and the *y*‐axis represents the square value of the correlation coefficient between connectivity *k* and *p* (*k*). The higher the square of the correlation coefficient, the closer the network approaches the distribution of a scale‐free network. Here, a soft threshold of 8 was selected. (B) The result graph of WGCNA power values for sarcopenia, where soft threshold (power) represents weight and the *y*‐axis represents average connectivity. (C) The cluster diagram of the sarcopenia module, the systematic clustering tree of genes and gene modules generated by the dynamic splicing method, where different colours represent different gene modules. (D) The results of correlation analysis between WGCNA module and disease status in sarcopenia. The number represents the correlation coefficient, and the number in parentheses below represents the significance *p* value. (E) Figure of WGCNA power values for osteoporosis. (F) The result chart of WGCNA power values for osteoporosis, where soft threshold (power) represents weight and the *y*‐axis represents average connectivity. (G) Cluster diagram of osteoporosis modules. (H) Correlation analysis results between osteoporosis WGCNA module and disease status.

According to the method, the WGCNA analysis was performed on differentially expressed genes in sarcopenia. First, a soft threshold of five was selected, as shown in Figure 2A,B. Second, based on clustering and dynamic pruning methods, the highly correlated genes were clustered into modules, which were then clustered. Modules with correlation coefficients greater than 0.75 and dissimilarity coefficients less than 0.25 are merged, resulting in a total of four modules, as shown in Figure 2C. Furthermore, by calculating the correlation between each module’s feature vector gene (which is the first principal component gene E of a specific module, representing the overall level of gene expression within the module) and phenotype (referring to two classification samples), the module was illustrated and *p* values were shown in Figure 2D. The blue module (388 genes; correlation coefficient |*r*| > 0.6, *p* < 0.05) was most significantly correlated with sarcopenia, whereas the magenta module (2533 genes; correlation coefficient |*r*| > 0.6, *p* < 0.05) was most significantly correlated with sarcopenia. Therefore, these two module genes were selected as the key module genes related to sarcopenia. Second, WGCNA was performed on the osteoporosis analysis dataset. First, a soft threshold of five was selected, as shown in Figure 2E,F. Second, based on clustering and dynamic pruning methods, the highly correlated genes were clustered into modules, which were then clustered. Modules with correlation coefficients greater than 0.75 and dissimilarity coefficients less than 0.25 are merged, resulting in a total of eight modules, as shown in Figure 2G. Furthermore, by calculating the correlation between each module’s feature vector gene (which is the first principal component gene E of a specific module, representing the overall level of gene expression within that module) and phenotype (referring to two classification samples), the modules with significant *p* value are plotted and displayed, as shown in Figure 2H. The blue module (1158 genes; correlation coefficient |*r*| > 0.5 and *p* < 0.05), black module (74 genes; correlation coefficient |*r*| > 0.5 and *p* < 0.05) and yellow module (424 genes; correlation coefficient |*r*| > 0.5, *p* < 0.05) were also significantly related to osteoporosis.

### 3.3 Identification of Co Susceptible Genes

**FIGURE 3:**
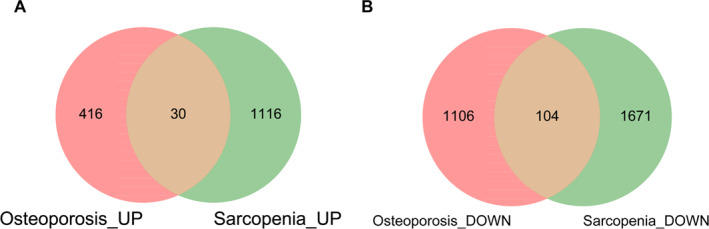
The results of the WGCNA power values for osteoporosis. (A) The Venn diagram showing the intersection of upregulated genes in sarcopenia‐related key modules and osteoporosis‐related key modules. (B) The Venn diagram showing the intersection of downregulated genes in sarcopenia‐related key modules and osteoporosis‐related key modules.

According to this method, the key module genes of these two diseases intersect. The genes in the key module of sarcopenia were divided into upregulation and downregulation groups, with 1146 upregulated genes and 1775 downregulated genes, respectively. The genes in the key module of osteoporosis were divided into upregulated and downregulated, with 446 upregulated genes and 1210 downregulated genes; therefore, the upregulated and downregulated genes in the key modules of the two diseases were intersected, as shown in Figure 3A,B. A total of 30 upregulated genes in common modules and 9104 downregulated genes in common modules were obtained.

### 3.4 GO and KEGG Pathway Analysis

GO function and KEGG pathway analyses were conducted on the shared module genes.

**FIGURE 4:**
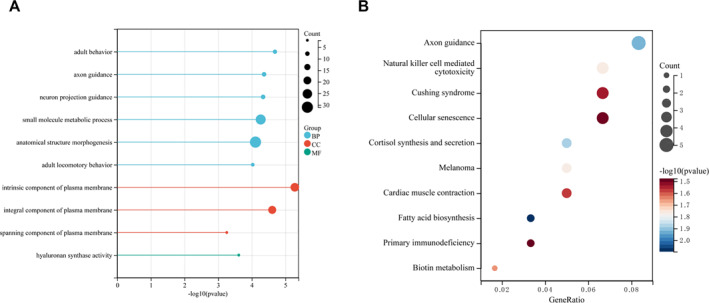
The Venn diagram of intersection of upregulated genes in key modules related to sarcopenia and osteoporosis. (A) GO enrichment map of common module genes. (B) KEGG enrichment map of common module genes.

To elucidate the biological functions of genes within the shared key modules associated with sarcopenia and osteoporosis, GO (Gene Ontology) functional and KEGG (Kyoto Encyclopedia of Genes and Genomes) pathway enrichment analyses were performed on the common module genes. Initially, a Venn diagram was constructed to identify the intersection of differentially expressed genes from the key modules related to both conditions. Specifically, the key module for sarcopenia contained 1146 upregulated and 1775 downregulated genes, whereas the key module for osteoporosis comprised 446 upregulated and 1210 downregulated genes. This comparative analysis revealed 30 upregulated and 104 downregulated genes common to both modules, as shown in Figure 4A,B. Subsequently, GO functional enrichment analysis of these common genes yielded 699 significant terms, with the results presented in Figure 4A. In parallel, KEGG pathway analysis identified 12 significantly enriched pathways, which are displayed in Figure 4B.

### 3.5 PPI Network Construction and Hub Gene Identification of Shared DEGs

**FIGURE 5:**
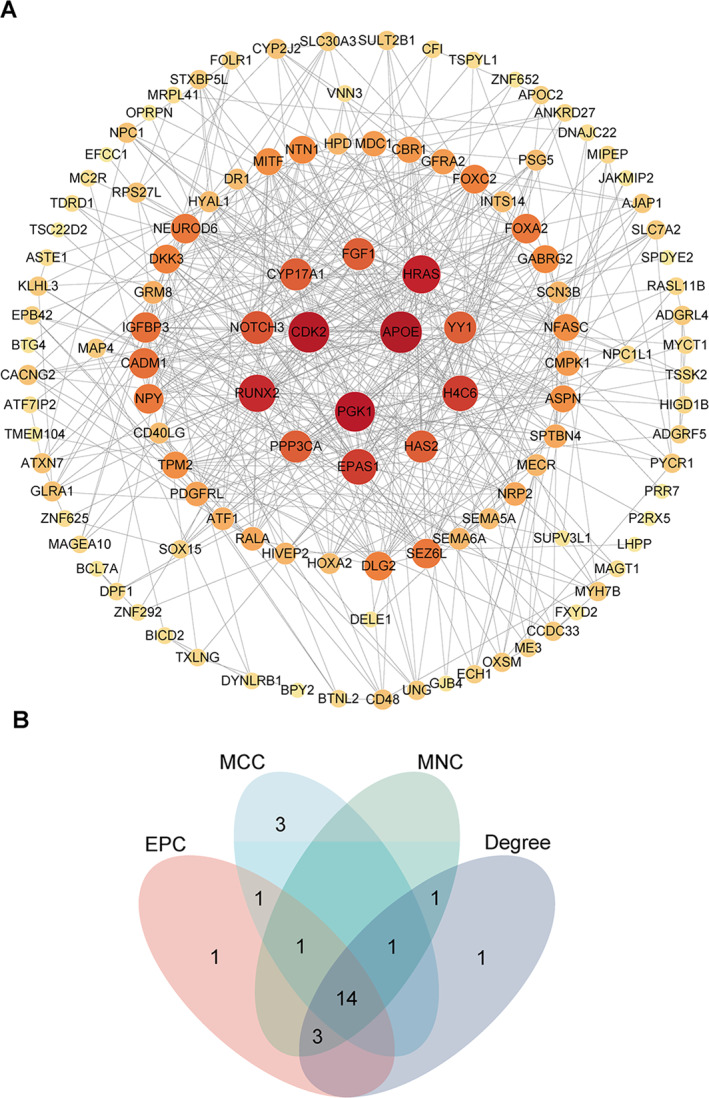
Hub genes identification of shared DEGs using PPI network and the expression of PGK1 in sarcopenia and osteoporosis. (A) PPI network of common disease‐related genes, with larger circles indicating more interacting proteins as the colour becomes orange as shown in panel (A). (B) Venn diagram of intersection of four indicators, as shown in panel (B).

Network analysis was performed on the proteins corresponding to the intersecting genes, as shown in Figure 5A. The plugin was then used to calculate indicators such as degree, MNC, MCC and EPC. The four indicators, degree, MNC, MCC and EPC, were arranged in descending order, and the top 20 genes were selected. The genes obtained from these four indicators intersected, resulting in a Venn diagram, as shown in Figure 5B and a total of 14 genes were obtained.

### 3.6 Efficacy Evaluation of Hub Genes

**FIGURE 6:**
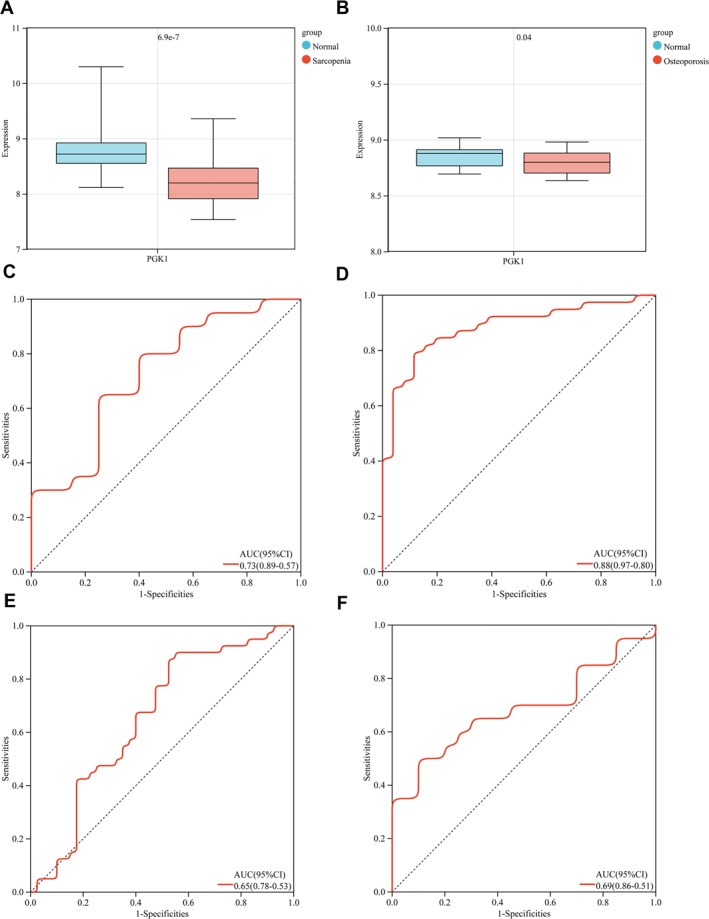
Evaluation of the efficacy of hub genes. (A) Box plot of expression level distribution of PGK1 in sarcopenia validation set samples, as shown in Figure 6A. (B) Box plot of expression level distribution of PGK1 in osteoporosis validation set samples, as shown in Figure 6B. (C) The ROC curves of the hub gene, PGK1 in the sarcopenia training set, as shown in Figure 6C. (D) The ROC curves of the hub gene, PGK1 in the sarcopenia validation set, as shown in Figure 6D. (E) The ROC curves of the hub gene, PGK1 in the osteoporosis training set, as shown in Figure 6E. (F) The ROC curves of the hub gene, PGK1 in the osteoporosis validation set, as shown in Figure 6F.

To verify the expression levels of key genes, the 14 key genes obtained from the above analysis and mining were mapped for differential analysis in the sarcopenia validation and osteoporosis validation sets. The results of the analysis are presented. A gene that was consistent with the upregulation in the training set and significant in both validation sets was selected, and one hub gene, PGK1, was obtained, as shown in Figure 6A,B. The ROC curves and the final hub gene expression across all datasets (training and validation sets) were plotted to evaluate the accuracy of diagnostic features using the pROC package in R language. The minimum range of ROC values for all genes was greater than 0.5, and the ROC values were generally above 0.6, indicating good accuracy (Figure 6C–F).

### 3.7 PPI Construction of Hub Gene

**FIGURE 7:**
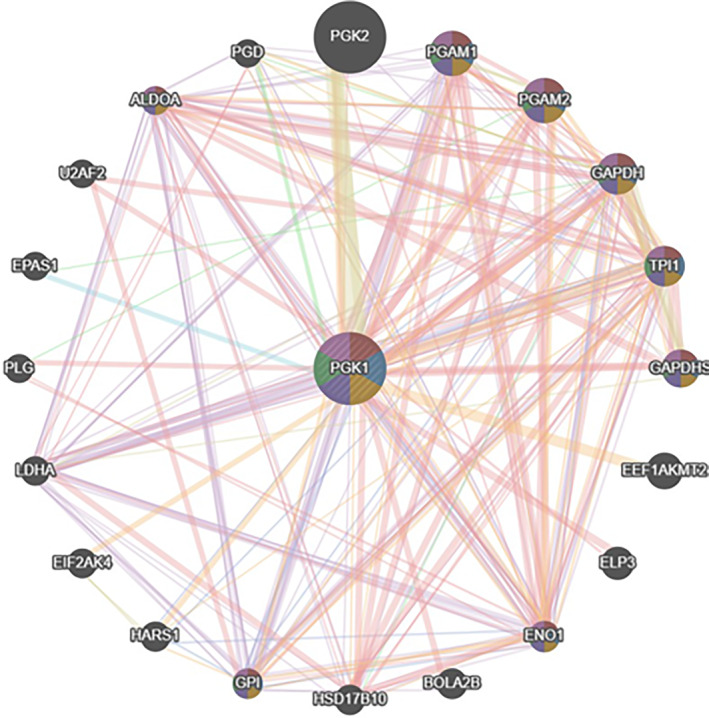
Constructing PPI for hub genes using the GeneMANIA database. The GeneMANIA database PPI analysis on the hub genes, including PGK2, PGAM1, ADOA, PGAM2, GAPDH, TPI1, PGK1, GAPDHS, LDHA, EEF1, PLG, U2AF2, PGD, EPAS1, EIF2AK4, AKMT2, HARS, BOLA2B, ENO1, ELP3, GPI, HSD17B10 and their interacting genes of PGK1, was conducted to predict colocalisation, shared protein domains, co‐expression, prediction and correlation pathways, as shown in this figure.

PPI analysis of the final hub genes was conducted using the GeneMANIA database, and their interacting genes were analysed to predict the correlation between colocalisation, shared protein domains, co‐expression, prediction and pathways (Figure 7).

### 3.8 Evaluation of Immune Cell and Stromal Cell Infiltration and Validation of Hub Gene Expression in HPA Database

**FIGURE 8:**
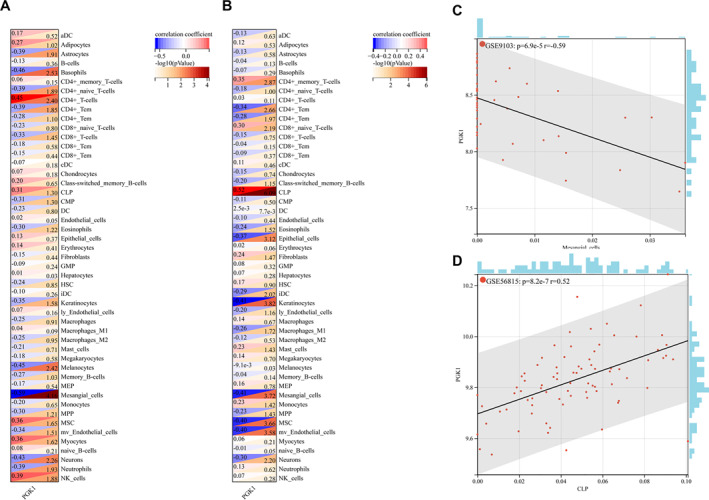
Evaluation of immune cell and stromal cell infiltration. (A) Heat map of the correlation between key genes and immune cells in sarcopenia. (B) Heat map of the correlation between key genes and immune cells in osteoporosis. (C) Scatter plot of Mesangial cells and PKG1 in sarcopenia. (D) Scatter plot of GLP and PKG1 in osteoporosis.

First, the online tool xCell was used to calculate the training dataset of stromal cell types and 64 immune cell types for sarcopenia and osteoporosis, respectively. The proportion of 64 immune cells in each sample was obtained for sarcopenia, and the proportion of 64 immune cells in each sample was obtained for osteoporosis. Subsequently, the correlation coefficients and *p* values between each key gene and each immune cell were calculated using the Spearman method, and correlation heatmaps were plotted. A heatmap of the correlation between key genes and immune cells in sarcopenia is shown in Figure 8A. A heatmap of the correlation between key genes and immune cells in osteoporosis is shown in Figure 8B. The most strongly associated immune cells and important genes in each of the two diseases were chosen for the scatter plot. The correlation between mesangial cells and PGK1 in sarcopenia was −0.59, whereas the correlation between common lymphoid progenitors (CLP) and PGK1 in osteoporosis was 0.52. The scatter plot of mesangial cells and PGK1 in sarcopenia is shown in Figure 8C, and the scatter plot of common lymphoid progenitors (CLP) and PGK1 in osteoporosis is shown in Figure 8D.

### 3.9 Validation of Hub Gene Expression in the HPA Database

**FIGURE 9:**
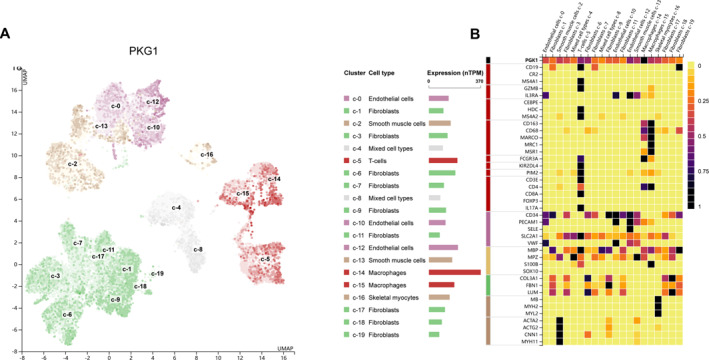
Validation of hub gene expression in HPA database. (A) Aggregation clusters of different single‐cell types in skeletal muscle. (B) Heat map of PKG1 and recognisable cell type markers expression in different single‐cell type clusters of skeletal muscle.

The expression of hub genes was validated in the Human Protein Atlas (HPA) database, which provides comprehensive single‐cell RNA sequencing (scRNA‐seq) and immunohistochemical (IHC) data. For PKG1, scRNA‐seq analysis revealed its predominant enrichment in skeletal muscle fibroblasts, a key cell type involved in muscle homoeostasis and regeneration, as shown in Figure 9A. (B) PKG1 expression was primarily localised to green‐coloured fibroblast clusters, indicating its specific role in this compartment. To further explore the relationship between PKG1 and skeletal muscle cell identity, Pearson correlation analysis was performed on single‐cell type clusters. The resulting heat map demonstrated a strong positive correlation between PKG1 expression and the abundance of skeletal muscle fibroblasts, highlighting its potential as a marker for this cell population, as shown in Figure 9B. These findings align with previous studies implicating PKG1 in muscle‐related biological processes, providing additional support for its functional relevance in skeletal muscle tissue.

### 3.10 Single Gene GSEA Analysis

**FIGURE 10:**
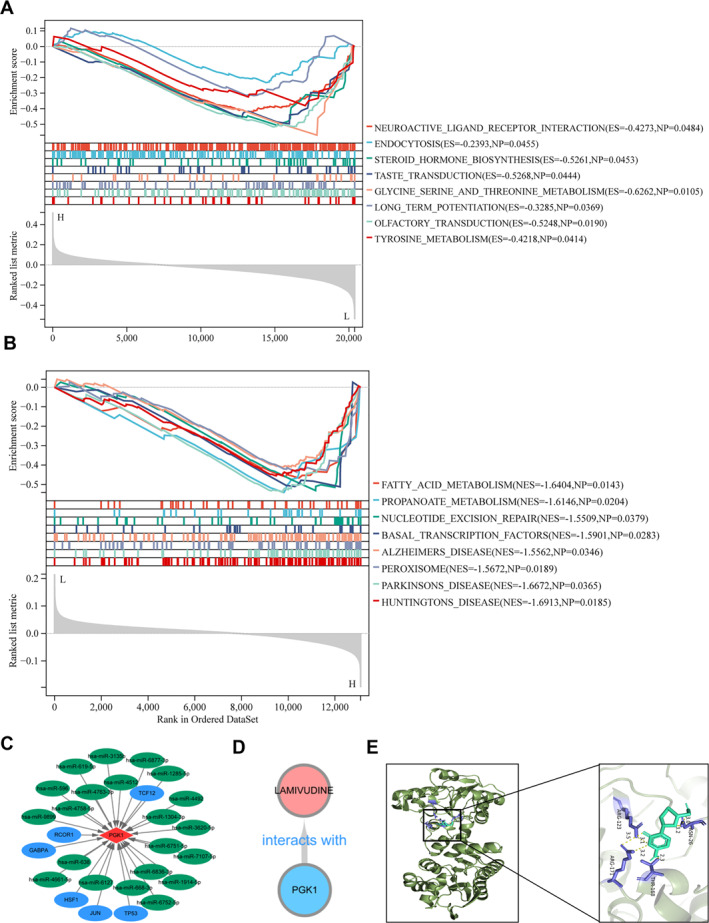
Single gene GSEA analysis, regulatory relationship between TF gene and miRNA gene co expression network and drug gene prediction. (A) GSEA enrichment analysis results of sarcopenia. (B) Osteoporosis GSEA enrichment analysis results. (C) TF target miRNA regulatory network. The diamond represents hub genes, the ellipse represents regulatory genes, the green represents miRNA and the blue represents TF. (D) Shows the network relationship between small molecule drugs and target genes. Blue represents hub genes and pink represents drugs. (E) Molecular docking showed that the vina score of LAMIVUDINE with PGK1 were mostly less than ‒5kcal/mol, The ASN‐26, THR‐168, ARG‐123, and ARG‐171 residues on the PGK1 receptor form hydrogen bonds with LAMIVUDINE. The dark green portion represents partial protein structures, the purple portion represents protein amino acid residues linked by hydrogen bonds, the green portion represents the chemical structure of the active substance, and the yellow portion represents hydrogen bonds linking protein residues by chemical substances, reflecting the binding capacity between LAMIVUDINE with PGK1, as shown in Figure 10E.

Based on the median expression of hub genes, the samples were divided into two groups for GSEA enrichment analysis to explore the enrichment of hub genes in the hallmarks (*p* < 0.05). The GSEA results of PGK1 gene expression in sarcopenia suggested 10 significantly negatively correlated pathways. The results for the activated and suppressed pathways are shown in Figure 10A. In addition, the GSEA results of PGK1 expression in sarcopenia suggest nine significantly negatively correlated pathways. The results of the activation and inhibition of some pathways are shown in Figure 10B.

### 3.11 Regulatory Relationship Between TF Gene and miRNA Gene Co Expression Network and Drug Gene Prediction

The hub gene was run to obtain a list of predicted miRNAs target regulatory relationship pairs in miRWalk 3.0 gene module. The list of predicted miRNAs target regulatory relationship pairs was run to select 21 miRNAs with regulatory relationships and free energy greater than 30. Subsequently, the hub gene was predicted for transcription factors (TF) using the Jaspar database, and six TF genes with regulatory relationships were screened to build a TF target miRNA network using Cytoscape software, as shown in Figure 10C. The Drug Bank database was used to predict drugs that interact with hub genes and to observe the interaction between drugs and genes, and LAMIVUDINE targeting PGK1 was to build a network relationship diagram, as shown in Figure 10D. Molecular docking showed that the vina score of LAMIVUDINE with PGK1 were mostly less than −5 kcal/mol, reflecting the binding capacity between LAMIVUDINE with PGK1, as shown in Figure 10D.

## 4 Discussion

According to epidemiological data, older people who live in the community and sustain fractures have varied incidences of osteosarcopenia [9]. Osteosarcoma diagnosis and treatment are now considered standard medical practices. To improve health outcomes, additional research is needed to uncover biomarkers that could lead to better diagnosis, risk assessment and therapy. A recent study found that older age, physical inactivity, low body mass index and increasing fat mass are risk factors for osteosarcopenia in women living in communities.

Osteoporosis and sarcopenia were significantly and inversely related to serum IGF1 and 25(OH)D. Despite adjusting for sex and BMI, no significant link was discovered between osteoporosis and sarcopenia or fragility fracture(s); nevertheless, sarcopenia and fracture(s) were significantly associated. Our findings could provide a health platform for the very old and suggest techniques to keep musculoskeletal diseases at bay [10].

A logistic regression analysis found that higher TSH, BAP and eGFR levels increased the likelihood of osteosarcopenia. In contrast, increased levels of BUN, K and 25(OH)D lowered the incidence of osteosarcopenia. Further research is needed to determine the influence of additional bone metabolism indicators [11]. The dysregulation of miRNAs offers the groundwork for future research that will result in efficient screening, monitoring or treatment approaches while also offering crucial hints to help comprehend the pathophysiology of osteosarcopenia. Database searches, meta‐analyses and screening techniques for lipid, amino acid and energy metabolism have all been used to find osteosarcopenia biomarkers. For instance, telomere shortening can be utilised alone or in conjunction with other biomarkers to monitor the development and course of osteoporosis [12]. A total of 9 shared dysregulation miRNAs, whose targets and functions are linked to the aetiology of osteosarcopenia, were discovered to be shared by osteoporosis and sarcopenia [13]. Nevertheless, the current research has trouble gathering metabolomics using different application techniques or detecting isomers. Moreover, small study cohorts and inadequate research may prevent the development of optimal biomarkers for diagnosis and treatment [14].

Determining the biomarkers of osteosarcopenia‐a combination of sarcopenia and osteopenia/osteoporosis‐is critical. ROCK1, a potential therapy target for postmenopausal osteoporosis, has been related to strong relationships with disease aetiology and immune infiltration. It has also been found to have an impact on cancer development, progression and prognosis [15]. A six‐hub gene‐based diagnostic paradigm for osteoporosis was developed using MYC, VEGFA, CSF1R, S100B, APOE and FGF13. PMAIP1 was found to be a promising biomarker for osteoporosis diagnosis and treatment [16, 17]. In addition, there was a considerable down‐regulation of EGFR, HMOX1, PGR, CXCL10, CCL5 and IL12B in OP patients [18]. Diagnostic biomarkers for sarcopenia may include PDHA1, DLAT, PDHB and NDUFC1 [19]. A series of diagnostic biomarkers (ARHGAP36, FAM171A1, GPCPD1, MT1X, ZNF415 and RXRG) were identified by LASSO analysis [20]. Using mRNA microarray analysis of five matched OS tissues, it was possible to identify differential genes in osteosarcoma by co‐expressing carboxypeptidase E, a prognostic biomarker, with osteoblastic genes [21]. The diagnostic performance of PGK1 was evaluated through receiver operating characteristic (ROC) curve analysis across sarcopenia and osteoporosis cohorts. In sarcopenia, PGK1 demonstrated area under the curve (AUC) values of 0.73 in the training set and 0.88 in the validation set, whereas in osteoporosis cohorts, the corresponding AUCs were 0.65 in the training set and 0.69 in the validation set. Although PGK1 achieved ROC‐AUC > 0.7 in sarcopenia diagnosis, its standalone diagnostic utility remains constrained by the inherent limitations of single‐gene biomarkers, as documented in prior biomarker studies. Notably, combinatorial approaches integrating PGK1 with mechanistically relevant genes to comprise biomarker panels to enhance predictive capacity, achieving AUC values exceeding 0.8. To optimise PGK1‐based diagnostic models, future investigations should prioritise multicenter cohort expansion and multimodal data integration incorporating essential clinical parameters (age, BMI) alongside molecular markers, thereby improving both diagnostic accuracy and clinical translatability. The glycolytic enzyme phosphoglycerate kinase 1 (PGK1) catalyses the transformation of 1,3‐diphosphoglycerate into 3‐phosphoglycerate and may act as a cofactor for polymerase alpha, which is secreted by tumour cells and plays a role in angiogenesis by breaking down disulfide bonds in the serine protease plasmin. This causes angiostatin, an inhibitor of tumour blood vessels, to be released. The capacity of PGK1 to carry out mechanistically unique tasks has led to its classification as a moonlighting protein. Haemolytic anaemia and neurological dysfunction are two of the many clinical characteristics linked to PGK1 deficiency. PGK1 is implicated in numerous biological processes, including those related to development and cancer, glucose metabolism, angiogenesis, the epithelial‐mesenchymal transition, mediated autophagy initiation, mitochondrial metabolism, DNA replication and repair [22]. PGK1 has several different roles, which makes its role in the genesis of cancer complex. Elevated levels of PGK1 expression outside the cell decrease angiogenesis, which in turn counteracts the aggressiveness of cancer. Low levels of PGK1 expression inside the cell promote the growth of tumour cells [23]. Targeting PGK1, a key glycolytic enzyme, presents a novel therapeutic strategy for keloids by suppressing the Warburg effect [24]. PGK1 downregulation reduces ATP production in fibroblasts, thereby impairing collagen synthesis (e.g., COL1A1 expression) and extracellular matrix remodelling—crucial processes for tissue repair [25]. Importantly, PGK1 inhibition diminishes keloid fibroblast proliferation, migration, invasion and type I collagen production through glycolysis suppression. The genetic deletion or pharmacological suppression of PGK1 significantly suppresses the progression and expansion of gastric cancer‐derived peritoneal metastases, directly implicating PGK1 in driving this pathological process. Furthermore, PGK1 upregulates the expression of CXCR4 and CXCL12—molecular mediators linked to metastatic behaviour that facilitate the tissue‐specific recruitment of cancer cells. These findings position PGK1, along with its regulatory networks and downstream effectors, as potential therapeutic targets for both inhibiting peritoneal metastasis formation and potentiating the tumoricidal activity of conventional chemotherapeutic agents [26]. Metabolic reprogramming is regulated by the mitochondrial circRNA for translocating PGK1, or mcPGK1, which promotes glycolysis and inhibits mitochondrial oxidative phosphorylation through the PDK1‐PDH axis, and its absence may lead to ROS accumulation and cellular senescence, thereby affecting the normal physiological functions of fibroblasts [27]. This modifies the intracellular concentrations of lactate and α‐ketoglutarate, which regulate the activation of Wnt/β‐catenin and the self‐renewal of liver TIC. PGK1 can affect osteogenic differentiation by regulating β—catenin stability, or indirectly inhibit Wnt signalling through GSK3β phosphorylation, which may be closely related to the development and maintenance of the skeletal system [28]. Furthermore, through TOM40 connections, mcPGK1 stimulates PGK1 mitochondrial import, which reprograms metabolism via the PGK1–PDK1–PDH axis from oxidative phosphorylation to glycolysis. Prescription antagonist terazosin (TZ) activates PGK1, which can break down abnormal protein aggregates by autophagy and dissolving them with elevated ATP levels when activated in the cytosol [29]. Neurite outgrowth is promoted by the extracellular PGK1’s interaction with the neuronal membrane receptor Enolase‐2, which lowers P38/Limk1/Cofilin signalling [30]. Potential PGK1 activators that could successfully mitigate apoptosis caused by OGD/R‐induced neuronal cell damage and protect against PQ‐induced oxidative stress in the Drosophila model [31].

According to the enrichment analysis of PGK1 co‐expressed genes in lung adenocarcinoma, PGK1 may be connected to several immunological and inflammatory signalling pathways, metabolism, hypoxia, DNA synthesis, the cell cycle and PI3K/AKT. Moreover, PGK1 has been connected to the recruitment of several immune cells, such as neutrophils, macrophages and aDCs (dendritic cells). More significantly, immune‐suppressive cells such as M2 macrophages, Tregs and fatigued T cells were shown to exhibit high levels of PGK1 [32].

PPGK1 represents a possible target for therapy. Neurodegenerative illness known as spinal muscular atrophy (SMA) is inherited recessively [33]. Central nervous system (CNS) dysfunction, myopathy and haemolytic anaemia were the three symptoms that the PGK1 mutation (PGK1 Galveston) displayed [34]. One of the main factors influencing phenotype in PGK deficiency is the degree of impaired glycolysis. Increased sensitivity to exertional rhabdomyolysis and multisystem involvement appears to be the outcomes of lower glycolytic capacity in PGK1 deficiency [35]. The findings of this study are further supported by recent clinical reports elucidating the multifaceted roles of PGK1 in human pathophysiology. Notably, Baba et al. [36]described a novel PGK1 mutation (p.S62N) associated with both myopathic and haemolytic manifestations, demonstrating the enzyme’s critical role in energy metabolism across muscular and haematopoietic systems. This discovery aligns with our observation of PGK1 downregulation in musculoskeletal tissues, suggesting conserved metabolic pathways may underlie both sarcopenia and osteoporosis. Furthermore, the phenotypic spectrum of PGK1 deficiency was remarkably expanded by Gutierrez et al. [34], who identified a paediatric case presenting with the triad of haemolytic anaemia, neurological dysfunction and myopathy, emphasising PGK1’s systemic regulatory functions. Importantly, Zhou et al. [37] recently extended these observations through an atypical PGK1 deficiency case featuring seizure manifestations alongside mild haemolytic anaemia, highlighting the neurological implications of PGK1 dysfunction that may parallel the central regulatory mechanisms observed in musculoskeletal degeneration. From a broader perspective, Di Lazzaro et al. [38] systematically reviewed X‐linked parkinsonian disorders, categorising PGK1 deficiency syndrome as a distinct entity within metabolic neurodegeneration disorders. This comprehensive analysis reinforces our hypothesis about PGK1’s pleiotropic effects across different tissue systems, potentially mediated through conserved glycolytic regulation and cellular energy homoeostasis mechanisms.

Mechanistically, PGK1 serves as a pivotal metabolic regulator in musculoskeletal tissues, orchestrating energy production through tissue‐specific mechanisms. Specifically, in skeletal muscle, PGK1 drives glycolytic flux to sustain the proliferation and differentiation of satellite cells—tissue‐resident stem cells critical for post‐injury regeneration. Experimental evidence strongly indicates that PGK1 depletion disrupts ATP‐dependent activation of these stem cells, significantly impairing post‐injury muscle repair [25]. Conversely, in bone tissue, PGK1 maintains mitochondrial bioenergetics within osteoblasts by modulating oxidative phosphorylation and membrane potential. Compelling studies demonstrate that reduced PGK1 activity triggers mitochondrial dysfunction—manifested through ROS accumulation and apoptotic signalling—ultimately compromising osteoblast‐mediated bone formation [28]. Collectively, this dual role highlights PGK1 as a molecular nexus coordinating glycolysis‐dependent muscle repair and oxidative metabolism‐dependent bone remodelling.

Therapeutically, PGK1 emerges as a promising target for musculoskeletal disorders. Particularly, its regulatory influence on both glycolytic (muscle) and mitochondrial (bone) pathways suggests context‐specific intervention strategies: agonism to enhance tissue repair in sarcopenia or osteoporosis, versus inhibition to mitigate pathological hyperactivation. However, future research should prioritise preclinical validation of PGK1 modulators and explore combinatorial approaches integrating clinical parameters (e.g., age and BMI) with molecular biomarkers to optimise diagnostic and therapeutic frameworks. Such efforts could potentially unlock novel treatments leveraging PGK1’s dual metabolic roles to restore musculoskeletal integrity.

Notably, this finding suggests that PGK1 deficiency may influence bone metabolism by modulating energy production and redox balance in osteoblasts. Specifically, the study [36] clearly demonstrates that silencing PGK1 via miR‐4523 activates the Nrf2 signalling cascade, thereby protecting human osteoblasts from dexamethasone‐induced oxidative injury and apoptosis. Importantly, the link provided in the review is valid and supports the assertion that PGK1 deficiency may be relevant to glucocorticoid‐induced osteoporosis. Furthermore, another study found that metabolic pathways and oxidative stress regulation are critical in bone health, which directly aligns with the potential impact of PGK1 deficiency on osteoporosis.

Mechanistically, the assertion that PGK1 deficiency disrupts glycolysis‐driven ATP production and forces metabolic adaptation towards mitochondrial oxidative phosphorylation is convincingly supported by the study by Chen et al. [39]. This pivotal work highlights the role of PGK1 in regulating energy homoeostasis in osteoblasts. Moreover, the observation that this metabolic shift could impair osteoblast differentiation and bone formation by depleting intermediates required for matrix mineralisation is strongly corroborated by the findings [26, 40]. Of particular significance, the claim that PGK1 dysfunction exacerbates oxidative stress due to impaired NAD+ regeneration is corroborated [40]. This landmark study shows that PGK1 deficiency leads to ROS accumulation and Nrf2 pathway activation, ultimately suggesting PGK1 deficiency may impact bone formation and resorption by influencing osteoblast energy metabolism and redox balance. These collective findings solidify PGK1’s role in glycolysis and its regulation of oxidative stress are essential for maintaining bone homoeostasis.

Building on this evidence, this study demonstrated [41] that miR‐4523‐mediated silencing of PGK1 protects osteoblasts from glucocorticoid (dexamethasone)‐induced oxidative damage and apoptosis by activating the Nrf2 antioxidant signalling pathway. Mechanistically speaking, PGK1 inhibition in osteoblasts modulates glucose metabolism and enhances redox homoeostasis, thereby establishing a potential link between dysregulated PGK1 activity and glucocorticoid‐induced osteoporosis. As a glycolytic rate‐limiting enzyme, PGK1 dysfunction may disrupt energy metabolism and redox balance in osteoblasts, consequently impairing bone formation or promoting excessive resorption. Notably, the Nrf2 pathway activation via PGK1 silencing aligns with metabolic‐oxidative stress regulation of bone homoeostasis, providing compelling support for PGK deficiency as a contributor to osteoporosis pathogenesis [42]. Additionally, glycolysis reprogramming (e.g., altered PGK activity) may indirectly influence osteoclast differentiation, thereby further cementing PGK’s involvement in bone remodelling dynamics [43].

Taken together, a total of 14 genes are identified by this bioinformatic investigation as hub genes for the co‐occurrence of osteoporosis and sarcopenia, with PGK1 standing out as the most strongly down‐regulated gene in both conditions. These findings position PGK1 as a potential common diagnostic and therapeutic target for sarcopenia and osteoporosis. Clinically relevant, the discovery of LAMIVUDINE as a PGK1‐targeting small molecule is particularly promising, especially given single‐cell evidence demonstrating PGK1’s predominant enrichment in skeletal muscle fibroblasts—a cellular population critically involved in musculoskeletal tissue homoeostasis.

## Author Contributions

**Kun Zhang:** conceptualization. **Hailong Li:** conceptualization, formal analysis, project administration. **Xinhong Chen:** data curation, resources. **Ping Tang:** data curation, resources. **Meng Wang:** methodology, visualization, **Chunting Yang:** investigation, methodology. **Rong Su:** software, visualization. **Xiaqin Gao:** data curation, formal analysis, methodology. **Fan Zhang:** formal analysis. **Juan Han:** investigation, writing – review and editing.

## Ethics Statement

The authors have nothing to report.

## Consent

All the authors have agreed to the publication of this paper.

## Conflicts of Interest

The authors declare no conflicts of interest.

## Data Availability

All data are available when requested by the readers from corresponding author.

## References

[1] V. Gopinath , “Osteoporosis,” Medical Clinics of North America 107, no. 2 (2023): 213–225, 10.1016/j.mcna.2022.10.013. 36759092

[2] A. J. Cruz‐Jentoft and A. A. Sayer , “Sarcopenia,” Lancet 393, no. 10191 (2019): 2636–2646, 10.1016/s0140-6736(19)31138-9. 31171417

[3] J. Xu , C. S. Wan , K. Ktoris , E. M. Reijnierse , and A. B. Maier , “Sarcopenia Is Associated With Mortality in Adults: A Systematic Review and Meta‐Analysis,” Gerontology 68, no. 4 (2022): 361–376, 10.1159/000517099. 34315158

[4] I. R. Reid , “A Broader Strategy for Osteoporosis Interventions,” Nature Reviews Endocrinology 16, no. 6 (2020): 333–339, 10.1038/s41574-020-0339-7. 32203407

[5] A. Tournadre , G. Vial , F. Capel , M. Soubrier , and Y. J. J. Boirie , “Sarcopenia,” Joint Bone Spine 86, no. 3 (2019): 309–314, 10.1016/j.jbspin.2018.08.001. 30098424

[6] Y. Shuai and S. C. Larsson , “Epidemiology of Sarcopenia: Prevalence, Risk Factors, and Consequences,” Metabolism—Clinical and Experimental 144 (2023): 155533, 10.1016/j.metabol.2023.155533. 36907247

[7] L. Chao , L. Ningyuan , X. Yu , Z. Ziyue , X. Tao , and L. Hui , “Osteoporosis and Sarcopenia‐Related Traits: A Bi‐Directional Mendelian Randomization Study,” Frontiers in Endocrinology 13 (2022): 975647, 10.3389/fendo.2022.975647. 36187130 PMC9515352

[8] D. J. N. Papandreou , K. Papadimitriou , G. Voulgaridou , et al., “Exercise and Nutrition Impact on Osteoporosis and Sarcopenia—The Incidence of Osteosarcopenia: A Narrative Review,” Nutrients 13, no. 12 (2021): 4499, 10.3390/nu13124499. 34960050 PMC8705961

[9] B. Kirk , J. Zanker , and G. Duque , “Osteosarcopenia: Epidemiology, Diagnosis, and Treatment‐Facts and Numbers,” Journal of Cachexia, Sarcopenia and Muscle 11, no. 3 (2020): 609–618, 10.1002/jcsm.12567. 32202056 PMC7296259

[10] R. Hata , K. Miyamoto , Y. Abe , et al., “Osteoporosis and Sarcopenia Are Associated With Each Other and Reduced IGF1 Levels Are a Risk for Both Diseases in the Very Old Elderly,” Bone 166 (2023): 116570, 10.1016/j.bone.2022.116570. 36182103

[11] T. Inoue , A. Shimizu , K. Murotani , et al., “Exploring Biomarkers of Osteosarcopenia in Older Adults Attending a Frailty Clinic,” Experimental Gerontology 172 (2023): 112047, 10.1016/j.exger.2022.112047. 36509299

[12] F. Kakridonis , S. G. Pneumatikos , E. Vakonaki , et al., “Telomere Length as a Predictive Biomarker in Osteoporosis (Review),” Biomedical Reports 19, no. 5 (2023): 87, 10.3892/br.2023.1669. 37881605 PMC10594068

[13] F. Salamanna , D. Contartese , A. Ruffilli , et al., “Sharing Circulating Micro‐RNAs Between Osteoporosis and Sarcopenia: A Systematic Review,” Life (Basel) 13, no. 3 (2023): 602, 10.3390/life13030602. 36983758 PMC10051676

[14] X. Zhao , S. Patil , F. Xu , X. Lin , and A. Qian , “Role of Biomolecules in Osteoclasts and Their Therapeutic Potential for Osteoporosis,” Biomolecules 11, no. 5 (2021): 747, 10.3390/biom11050747. 34067783 PMC8156890

[15] B. Lai , H. Jiang , Y. Gao , and X. Zhou , “Identification of ROCK1 as a Novel Biomarker for Postmenopausal Osteoporosis and Pan‐Cancer Analysis,” Aging (Albany NY) 15, no. 17 (2023): 8873–8907, 10.18632/aging.205004. 37683138 PMC10522383

[16] T. Li , J. Yuan , P. Xu , et al., “PMAIP1, a Novel Diagnostic and Potential Therapeutic Biomarker in Osteoporosis,” Aging (Albany NY) 16, no. 4 (2024): 3694–3715, 10.18632/aging.205553. 38372699 PMC10929792

[17] Y. Zhao , J. Yan , Y. Zhu , Z. Han , T. Li , and L. Wang , “A Novel Prognostic 6‐Gene Signature for Osteoporosis,” Frontiers in Endocrinology 13 (2022): 968397, 10.3389/fendo.2022.968397. 36213260 PMC9533022

[18] J. Minbo , C. Feng , H. Wen , et al., “Up‐Regulated and Hypomethylated Genes Are Causative Factors and Diagnostic Markers of Osteoporosis,” American Journal of Translational Research 15, no. 10 (2023): 6042–6057. https://pubmed.ncbi.nlm.nih.gov/37969207/. 37969207 PMC10641362

[19] Y. Zhu , X. Chen , S. Geng , et al., “Identification of the Cuproptosis‐Related Hub Genes and Therapeutic Agents for Sarcopenia,” Frontiers in Genetics 14 (2023): 1136763, 10.3389/fgene.2023.1136763. 37007946 PMC10063920

[20] S. Lin , M. Ling , C. Chen , X. Cai , F. Yang , and Y. Fan , “Screening Potential Diagnostic Biomarkers for Age‐Related Sarcopenia in the Elderly Population by WGCNA and LASSO,” BioMed Research International 2022, no. 1 (2022): 7483911, 10.1155/2022/7483911. 36147639 PMC9489359

[21] D. Chen , B. Wan , Y. Cheng , et al., “Carboxypeptidase E Is a Prognostic Biomarker Co‐Expressed With Osteoblastic Genes in Osteosarcoma,” PeerJ 11 (2023): e15814, 10.7717/peerj.15814. 37663298 PMC10474831

[22] K. Zhang , L. Sun , and Y. Kang , “Regulation of Phosphoglycerate Kinase 1 and Its Critical Role in Cancer,” Cell Communication and Signaling 21 (2023): 240, 10.1186/s12964-023-01256-4. 37723547 PMC10506215

[23] Y. He , Y. Luo , D. Zhang , et al., “PGK1‐Mediated Cancer Progression and Drug Resistance,” American Journal of Cancer Research 9, no. 11 (2019): 2280–2302. https://pubmed.ncbi.nlm.nih.gov/31815035/. 31815035 PMC6895440

[24] P. Wang , Q. Wang , X. Yang , et al., “Targeting the Glycolytic Enzyme PGK1 to Inhibit the Warburg Effect: A New Strategy for Keloid Therapy,” Plastic and Reconstructive Surgery 151, no. 6 (2023): 970e–980e, 10.1097/prs.0000000000010137. 36728674

[25] C. Huang , C. Blecker , L. Chen , et al., “Integrating Identification and Targeted Proteomics to Discover the Potential Indicators of Postmortem Lamb Meat Quality,” Meat Science 199 (2023): 109126, 10.1016/j.meatsci.2023.109126. 36736126

[26] R. B. Wilson , W. Solass , R. Archid , F. J. Weinreich , A. Königsrainer , and M. A. Reymond , “Resistance to Anoikis in Transcoelomic Shedding: The Role of Glycolytic Enzymes,” Pleura Peritoneum 4, no. 1 (2019): 20190003, 10.1515/pp-2019-0003. 31198853 PMC6545877

[27] Z. Chen , Q. He , T. Lu , et al., “mcPGK1‐Dependent Mitochondrial Import of PGK1 Promotes Metabolic Reprogramming and Self‐Renewal of Liver TICs,” Nature Communications 14, no. 1 (2023): 1121, 10.1038/s41467-023-36651-5. PMC9971191 36849569

[28] J. Liang , X.‐y Zhang , Y.‐F. Zhen , et al., “PGK1 Depletion Activates Nrf2 Signaling to Protect Human Osteoblasts From Dexamethasone,” Cell Death & Disease 10, no. 12 (2019): 888, 10.1038/s41419-019-2112-1. 31767834 PMC6877585

[29] H. Chen , Y. Li , J. Gao , Q. Cheng , L. Liu , and R. Cai , “Activation of Pgk1 Results in Reduced Protein Aggregation in Diverse Neurodegenerative Conditions,” Molecular Neurobiology 60, no. 9 (2023): 5090–5101, 10.1007/s12035-023-03389-6. 37249790

[30] C. Y. Fu , H. Y. Chen , C. Y. Lin , S. J. Chen , J. C. Sheu , and H. J. Tsai , “Extracellular Pgk1 Interacts Neural Membrane Protein Enolase‐2 to Improve the Neurite Outgrowth of Motor Neurons,” Communications Biology 6, no. 1 (2023): 849, 10.1038/s42003-023-05223-0. 37582937 PMC10427645

[31] S. J. Qiang , Y. Q. Shi , T. Y. Wu , et al., “The Discovery of Novel PGK1 Activators as Apoptotic Inhibiting and Neuroprotective Agents,” Frontiers in Pharmacology 13 (2022): 877706, 10.3389/fphar.2022.877706. 35387336 PMC8978560

[32] Y. Yang , H. Cui , D. Li , et al., “Prognosis and Immunological Characteristics of PGK1 in Lung Adenocarcinoma: A Systematic Analysis,” Cancers (Basel) 14, no. 21 (2022): 5228, 10.3390/cancers14215228. 36358653 PMC9653683

[33] D. Gonzalez , C. Vásquez‐Doorman , A. Luna , and M. L. Allende , “Modeling Spinal Muscular Atrophy in Zebrafish: Current Advances and Future Perspectives,” International Journal of Molecular Sciences 25, no. 4 (2024): 1962, 10.3390/ijms25041962. 38396640 PMC10888324

[34] E. Gutierrez , M. G. Bayes , J. Mallick , L. Dell’osso , K. A. Lyapichev , and A. Muthukumar , “Recognition of a Novel Variant of Phosphoglycerate Kinase 1 Deficiency PGK1 Galveston (c.472G > C) in a Child With Hemolytic Anemia, Neurologic Dysfunction and Myopathy,” Pediatric Hematology & Oncology 40 (2023): 76–85, 10.1080/08880018.2022.2072987. 35608390

[35] J. Vissing , H. O. Akman , J. Aasly , et al., “Level of Residual Enzyme Activity Modulates the Phenotype in Phosphoglycerate Kinase Deficiency,” Neurology 91, no. 11 (2018): e1077–e1082, 10.1212/wnl.0000000000006165. 30111548

[36] K. Baba , T. Fukuda , M. Furuta , et al., “A Mild Clinical Phenotype With Myopathic and Hemolytic Forms of Phosphoglycerate Kinase Deficiency (PGK Osaka): A Case Report and Literature Review,” Internal Medicine 61, no. 23 (2022): 3589–3594, 10.2169/internalmedicine.9221-21. 35527021 PMC9790788

[37] X. Zhou , Q. Liu , and M. Huang , “An Atypical Case of Phosphoglycerate Kinase Deficiency With a Novel PGK1 Variant,” Seizure 117 (2024): 161–163, 10.1016/j.seizure.2024.02.010. 38432079

[38] G. Di Lazzaro , F. Magrinelli , C. Estevez‐Fraga , E. M. Valente , A. Pisani , and K. P. Bhatia , “X‐Linked Parkinsonism: Phenotypic and Genetic Heterogeneity,” Movement Disorders 36, no. 7 (2021): 1511–1525, 10.1002/mds.28565. 33960519

[39] J. C. Bertels , G. He , and F. Long , “Metabolic Reprogramming in Skeletal Cell Differentiation,” Bone Research 12, no. 1 (2024): 57, 10.1038/s41413-024-00374-0. 39394187 PMC11470040

[40] J. Han , Y. H. Kim , and S. Han , “Increased Oxidative Phosphorylation Through Pyruvate Dehydrogenase Kinase 2 Deficiency Ameliorates Cartilage Degradation in Mice With Surgically Induced Osteoarthritis,” Experimental & Molecular Medicine 57 (2025): 390–401, 10.1038/s12276-025-01400-9. 39894827 PMC11873213

[41] J. Q. Liang , Z. T. Zhou , L. Bo , H. N. Tan , J. H. Hu , and M. S. Tan , “Phosphoglycerate Kinase 1 Silencing by a Novel microRNA microRNA‐4523 Protects Human Osteoblasts From Dexamethasone Through Activation of Nrf2 Signaling Cascade,” Cell Death & Disease 12, no. 11 (2021): 964, 10.1038/s41419-021-04250-1. 34667156 PMC8526604

[42] Z. Feng , Y. Ou , and L. Hao , “The Roles of Glycolysis in Osteosarcoma,” Frontiers in Pharmacology 13 (2022): 950886, 10.3389/fphar.2022.950886. 36059961 PMC9428632

[43] J. Chen , J. Q. Liang , Y. F. Zhen , L. Chang , Z. T. Zhou , and X. J. Shen , “DCAF1‐Targeting microRNA‐3175 Activates Nrf2 Signaling and Inhibits Dexamethasone‐Induced Oxidative Injury in Human Osteoblasts,” Cell Death & Disease 12, no. 11 (2021): 1024, 10.1038/s41419-021-04300-8. 34716304 PMC8556244

